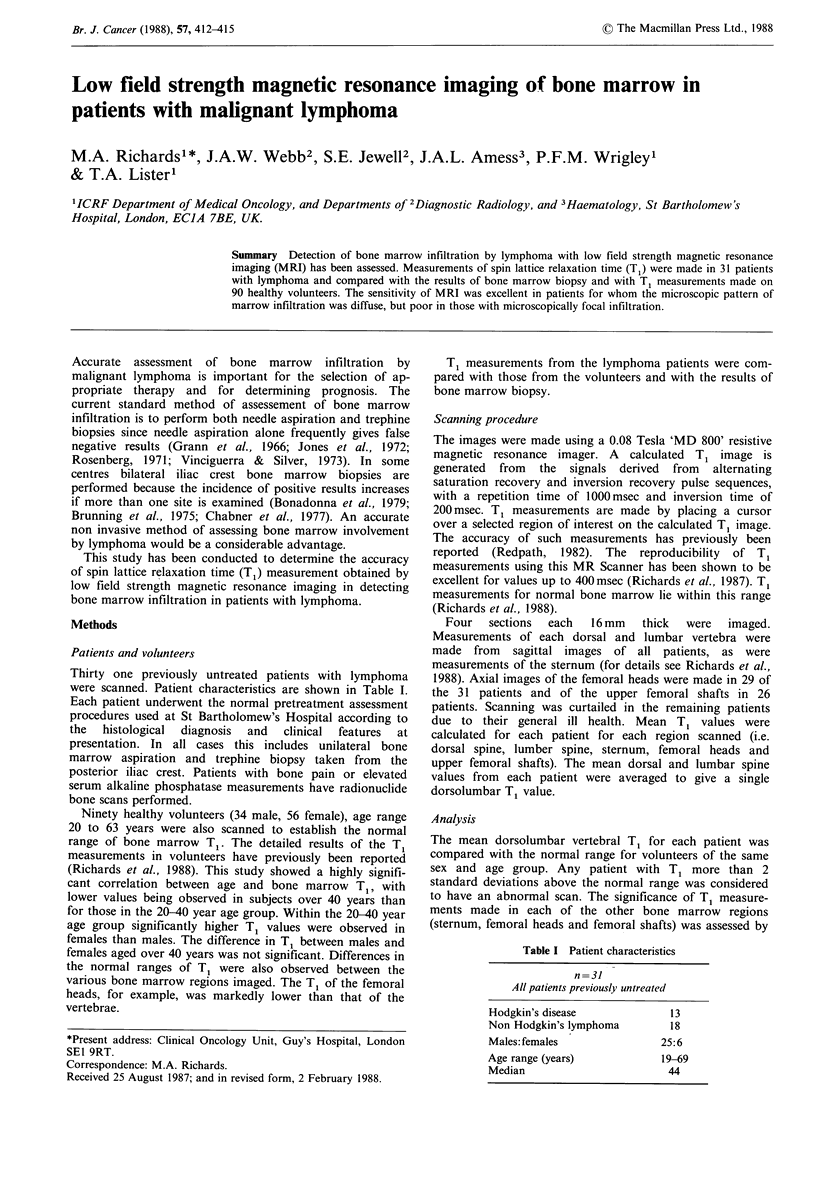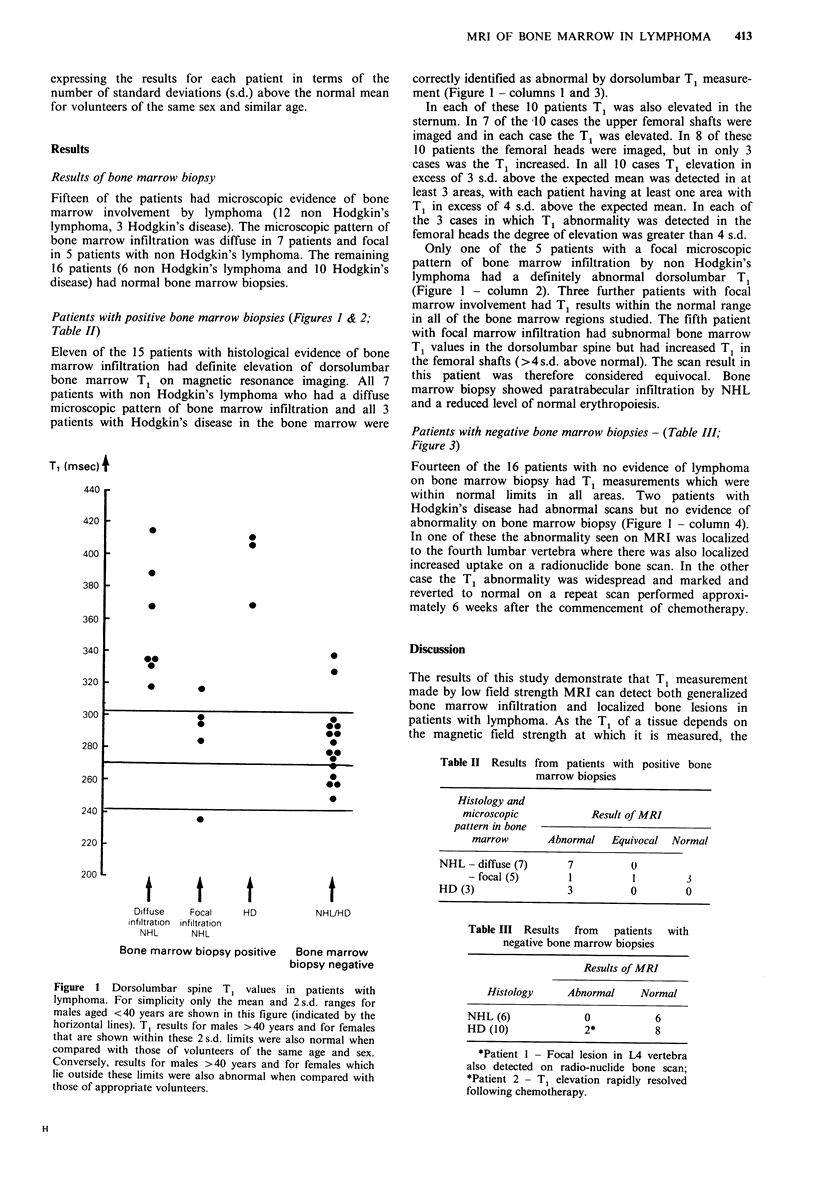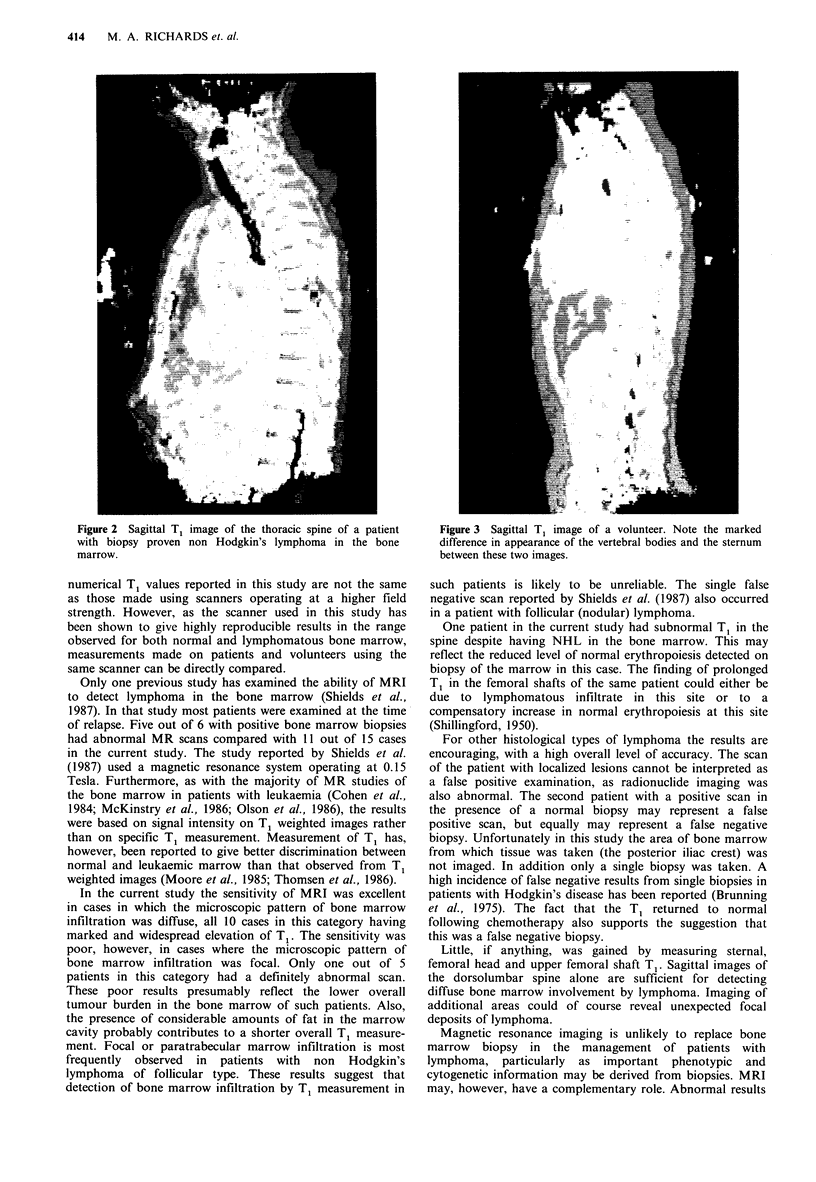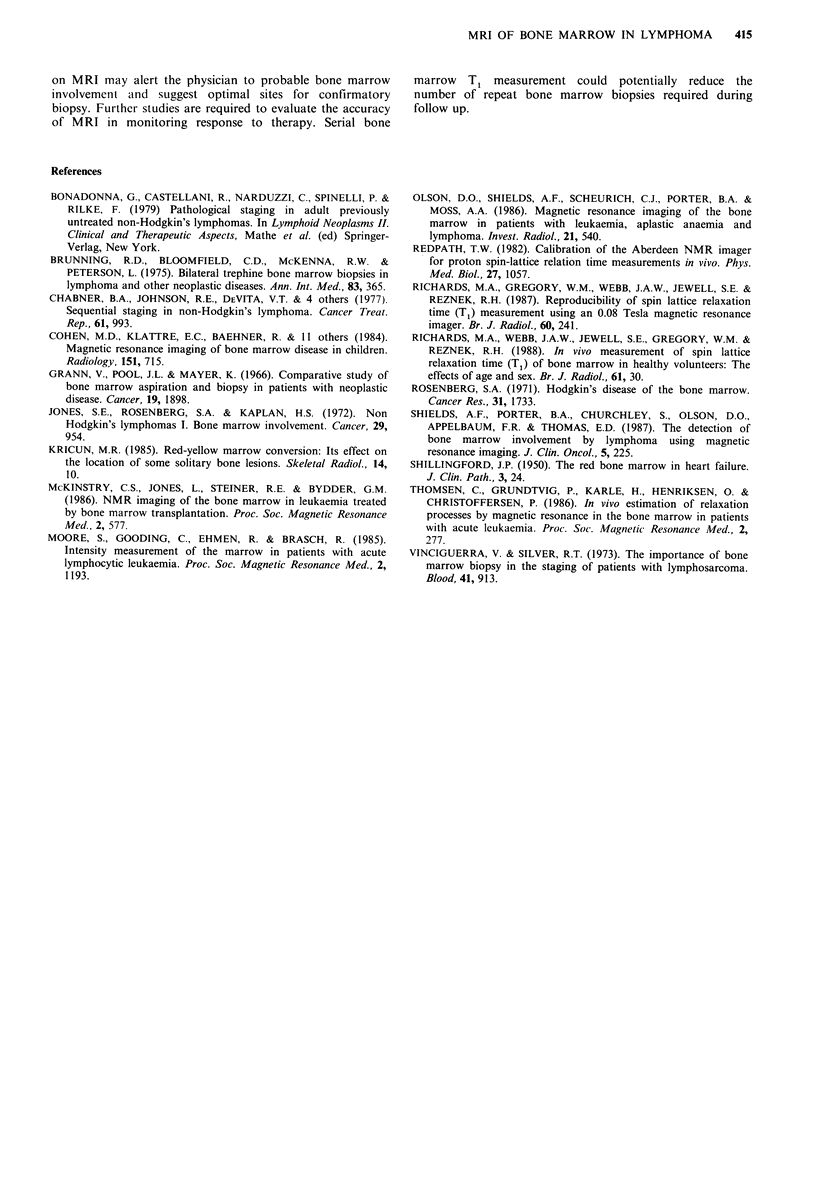# Low field strength magnetic resonance imaging of bone marrow in patients with malignant lymphoma.

**DOI:** 10.1038/bjc.1988.93

**Published:** 1988-04

**Authors:** M. A. Richards, J. A. Webb, S. E. Jewell, J. A. Amess, P. F. Wrigley, T. A. Lister

**Affiliations:** ICRF Department of Medical Oncology, St Bartholomew's Hospital, London, UK.

## Abstract

**Images:**


Br. J. Cancer (1988), 57, 412–415
© The Macmillan Press Ltd., 1988

### Low field strength magnetic resonance imaging of bone marrow in patients with malignant lymphoma

M.A. Richards[1]*, J.A.W. Webb[2], S.E. Jewell[2], J.A.L. Amess[3], P.F.M. Wrigley[1] & T.A. Lister[1]

[1]*ICRF Department of Medical Oncology, and Departments of* [2]*Diagnostic Radiology, and* [3]*Haematology, St Bartholomew's Hospital, London, EC1A 7BE, UK.*

**Summary** Detection of bone marrow infiltration by lymphoma with low field strength magnetic resonance imaging (MRI) has been assessed. Measurements of spin lattice relaxation time ($T_1$) were made in 31 patients with lymphoma and compared with the results of bone marrow biopsy and with $T_1$ measurements made on 90 healthy volunteers. The sensitivity of MRI was excellent in patients for whom the microscopic pattern of marrow infiltration was diffuse, but poor in those with microscopically focal infiltration.

Accurate assessment of bone marrow infiltration by malignant lymphoma is important for the selection of appropriate therapy and for determining prognosis. The current standard method of assessement of bone marrow infiltration is to perform both needle aspiration and trephine biopsies since needle aspiration alone frequently gives false negative results (Grann *et al.*, 1966; Jones *et al.*, 1972; Rosenberg, 1971; Vinciguerra & Silver, 1973). In some centres bilateral iliac crest bone marrow biopsies are performed because the incidence of positive results increases if more than one site is examined (Bonadonna *et al.*, 1979; Brunning *et al.*, 1975; Chabner *et al.*, 1977). An accurate non invasive method of assessing bone marrow involvement by lymphoma would be a considerable advantage.

This study has been conducted to determine the accuracy of spin lattice relaxation time ($T_1$) measurement obtained by low field strength magnetic resonance imaging in detecting bone marrow infiltration in patients with lymphoma.

### Methods

### *Patients and volunteers*

Thirty one previously untreated patients with lymphoma were scanned. Patient characteristics are shown in Table I. Each patient underwent the normal pretreatment assessment procedures used at St Bartholomew's Hospital according to the histological diagnosis and clinical features at presentation. In all cases this includes unilateral bone marrow aspiration and trephine biopsy taken from the posterior iliac crest. Patients with bone pain or elevated serum alkaline phosphatase measurements have radionuclide bone scans performed.

Ninety healthy volunteers (34 male, 56 female), age range 20 to 63 years were also scanned to establish the normal range of bone marrow $T_1$. The detailed results of the $T_1$ measurements in volunteers have previously been reported (Richards *et al.*, 1988). This study showed a highly significant correlation between age and bone marrow $T_1$, with lower values being observed in subjects over 40 years than for those in the 20–40 year age group. Within the 20–40 year age group significantly higher $T_1$ values were observed in females than males. The difference in $T_1$ between males and females aged over 40 years was not significant. Differences in the normal ranges of $T_1$ were also observed between the various bone marrow regions imaged. The $T_1$ of the femoral heads, for example, was markedly lower than that of the vertebrae.

*Present address: Clinical Oncology Unit, Guy's Hospital, London SE1 9RT.
Correspondence: M.A. Richards.
Received 25 August 1987; and in revised form, 2 February 1988.

$T_1$ measurements from the lymphoma patients were compared with those from the volunteers and with the results of bone marrow biopsy.

### *Scanning procedure*

The images were made using a 0.08 Tesla 'MD 800' resistive magnetic resonance imager. A calculated $T_1$ image is generated from the signals derived from alternating saturation recovery and inversion recovery pulse sequences, with a repetition time of 1000 msec and inversion time of 200 msec. $T_1$ measurements are made by placing a cursor over a selected region of interest on the calculated $T_1$ image. The accuracy of such measurements has previously been reported (Redpath, 1982). The reproducibility of $T_1$ measurements using this MR Scanner has been shown to be excellent for values up to 400 msec (Richards *et al.*, 1987). $T_1$ measurements for normal bone marrow lie within this range (Richards *et al.*, 1988).

Four sections each 16 mm thick were imaged. Measurements of each dorsal and lumbar vertebra were made from sagittal images of all patients, as were measurements of the sternum (for details see Richards *et al.*, 1988). Axial images of the femoral heads were made in 29 of the 31 patients and of the upper femoral shafts in 26 patients. Scanning was curtailed in the remaining patients due to their general ill health. Mean $T_1$ values were calculated for each patient for each region scanned (i.e. dorsal spine, lumber spine, sternum, femoral heads and upper femoral shafts). The mean dorsal and lumbar spine values from each patient were averaged to give a single dorsolumbar $T_1$ value.

### *Analysis*

The mean dorsolumbar vertebral $T_1$ for each patient was compared with the normal range for volunteers of the same sex and age group. Any patient with $T_1$ more than 2 standard deviations above the normal range was considered to have an abnormal scan. The significance of $T_1$ measurements made in each of the other bone marrow regions (sternum, femoral heads and femoral shafts) was assessed by

**Table I** Patient characteristics

| *n=31* *All patients previously untreated* | |
|---|---|
| Hodgkin's disease | 13 |
| Non Hodgkin's lymphoma | 18 |
| Males: females | 25:6 |
| Age range (years) | 19–69 |
| Median | 44 |

expressing the results for each patient in terms of the number of standard deviations (s.d.) above the normal mean for volunteers of the same sex and similar age.

### Results

### *Results of bone marrow biopsy*

Fifteen of the patients had microscopic evidence of bone marrow involvement by lymphoma (12 non Hodgkin's lymphoma, 3 Hodgkin's disease). The microscopic pattern of bone marrow infiltration was diffuse in 7 patients and focal in 5 patients with non Hodgkin's lymphoma. The remaining 16 patients (6 non Hodgkin's lymphoma and 10 Hodgkin's disease) had normal bone marrow biopsies.

### *Patients with positive bone marrow biopsies (Figures 1 & 2; Table II)*

Eleven of the 15 patients with histological evidence of bone marrow infiltration had definite elevation of dorsolumbar bone marrow $T_1$ on magnetic resonance imaging. All 7 patients with non Hodgkin's lymphoma who had a diffuse microscopic pattern of bone marrow infiltration and all 3 patients with Hodgkin's disease in the bone marrow were correctly identified as abnormal by dorsolumbar $T_1$ measurement (Figure 1 – columns 1 and 3).

In each of these 10 patients $T_1$ was also elevated in the sternum. In 7 of the 10 cases the upper femoral shafts were imaged and in each case the $T_1$ was elevated. In 8 of these 10 patients the femoral heads were imaged, but in only 3 cases was the $T_1$ increased. In all 10 cases $T_1$ elevation in excess of 3 s.d. above the expected mean was detected in at least 3 areas, with each patient having at least one area with $T_1$ in excess of 4 s.d. above the expected mean. In each of the 3 cases in which $T_1$ abnormality was detected in the femoral heads the degree of elevation was greater than 4 s.d.

Only one of the 5 patients with a focal microscopic pattern of bone marrow infiltration by non Hodgkin's lymphoma had a definitely abnormal dorsolumbar $T_1$ (Figure 1 – column 2). Three further patients with focal marrow involvement had $T_1$ results within the normal range in all of the bone marrow regions studied. The fifth patient with focal marrow infiltration had subnormal bone marrow $T_1$ values in the dorsolumbar spine but had increased $T_1$ in the femoral shafts (>4 s.d. above normal). The scan result in this patient was therefore considered equivocal. Bone marrow biopsy showed paratrabecular infiltration by NHL and a reduced level of normal erythropoiesis.

**Figure 1** Dorsolumbar spine $T_1$ values in patients with lymphoma. For simplicity only the mean and 2 s.d. ranges for males aged <40 years are shown in this figure (indicated by the horizontal lines). $T_1$ results for males >40 years and for females that are shown within these 2 s.d. limits were also normal when compared with those of volunteers of the same age and sex. Conversely, results for males >40 years and for females which lie outside these limits were also abnormal when compared with those of appropriate volunteers.

### *Patients with negative bone marrow biopsies – (Table III; Figure 3)*

Fourteen of the 16 patients with no evidence of lymphoma on bone marrow biopsy had $T_1$ measurements which were within normal limits in all areas. Two patients with Hodgkin's disease had abnormal scans but no evidence of abnormality on bone marrow biopsy (Figure 1 – column 4). In one of these the abnormality seen on MRI was localized to the fourth lumbar vertebra where there was also localized increased uptake on a radionuclide bone scan. In the other case the $T_1$ abnormality was widespread and marked and reverted to normal on a repeat scan performed approximately 6 weeks after the commencement of chemotherapy.

### Discussion

The results of this study demonstrate that $T_1$ measurement made by low field strength MRI can detect both generalized bone marrow infiltration and localized bone lesions in patients with lymphoma. As the $T_1$ of a tissue depends on the magnetic field strength at which it is measured, the

**Table II** Results from patients with positive bone marrow biopsies

| *Histology and microscopic pattern in bone marrow* | *Result of MRI* | | |
|---|---|---|---|
| | *Abnormal* | *Equivocal* | *Normal* |
| NHL – diffuse (7) | 7 | 0 | |
| – focal (5) | 1 | 1 | 3 |
| HD (3) | 3 | 0 | 0 |

**Table III** Results from patients with negative bone marrow biopsies

| *Histology* | *Results of MRI* | |
|---|---|---|
| | *Abnormal* | *Normal* |
| NHL (6) | 0 | 6 |
| HD (10) | 2* | 8 |

*Patient 1 – Focal lesion in L4 vertebra also detected on radio-nuclide bone scan; *Patient 2 – $T_1$ elevation rapidly resolved following chemotherapy.

**Figure 2** Sagittal $T_1$ image of the thoracic spine of a patient with biopsy proven non Hodgkin's lymphoma in the bone marrow.

**Figure 3** Sagittal $T_1$ image of a volunteer. Note the marked difference in appearance of the vertebral bodies and the sternum between these two images.

numerical $T_1$ values reported in this study are not the same as those made using scanners operating at a higher field strength. However, as the scanner used in this study has been shown to give highly reproducible results in the range observed for both normal and lymphomatous bone marrow, measurements made on patients and volunteers using the same scanner can be directly compared.

Only one previous study has examined the ability of MRI to detect lymphoma in the bone marrow (Shields *et al.*, 1987). In that study most patients were examined at the time of relapse. Five out of 6 with positive bone marrow biopsies had abnormal MR scans compared with 11 out of 15 cases in the current study. The study reported by Shields *et al.* (1987) used a magnetic resonance system operating at 0.15 Tesla. Furthermore, as with the majority of MR studies of the bone marrow in patients with leukaemia (Cohen *et al.*, 1984; McKinstry *et al.*, 1986; Olson *et al.*, 1986), the results were based on signal intensity on $T_1$ weighted images rather than on specific $T_1$ measurement. Measurement of $T_1$ has, however, been reported to give better discrimination between normal and leukaemic marrow than that observed from $T_1$ weighted images (Moore *et al.*, 1985; Thomsen *et al.*, 1986).

In the current study the sensitivity of MRI was excellent in cases in which the microscopic pattern of bone marrow infiltration was diffuse, all 10 cases in this category having marked and widespread elevation of $T_1$. The sensitivity was poor, however, in cases where the microscopic pattern of bone marrow infiltration was focal. Only one out of 5 patients in this category had a definitely abnormal scan. These poor results presumably reflect the lower overall tumour burden in the bone marrow of such patients. Also, the presence of considerable amounts of fat in the marrow cavity probably contributes to a shorter overall $T_1$ measurement. Focal or paratrabecular marrow infiltration is most frequently observed in patients with non Hodgkin's lymphoma of follicular type. These results suggest that detection of bone marrow infiltration by $T_1$ measurement in such patients is likely to be unreliable. The single false negative scan reported by Shields *et al.* (1987) also occurred in a patient with follicular (nodular) lymphoma.

One patient in the current study had subnormal $T_1$ in the spine despite having NHL in the bone marrow. This may reflect the reduced level of normal erythropoiesis detected on biopsy of the marrow in this case. The finding of prolonged $T_1$ in the femoral shafts of the same patient could either be due to lymphomatous infiltrate in this site or to a compensatory increase in normal erythropoiesis at this site (Shillingford, 1950).

For other histological types of lymphoma the results are encouraging, with a high overall level of accuracy. The scan of the patient with localized lesions cannot be interpreted as a false positive examination, as radionuclide imaging was also abnormal. The second patient with a positive scan in the presence of a normal biopsy may represent a false positive scan, but equally may represent a false negative biopsy. Unfortunately in this study the area of bone marrow from which tissue was taken (the posterior iliac crest) was not imaged. In addition only a single biopsy was taken. A high incidence of false negative results from single biopsies in patients with Hodgkin's disease has been reported (Brunning *et al.*, 1975). The fact that the $T_1$ returned to normal following chemotherapy also supports the suggestion that this was a false negative biopsy.

Little, if anything, was gained by measuring sternal, femoral head and upper femoral shaft $T_1$. Sagittal images of the dorsolumbar spine alone are sufficient for detecting diffuse bone marrow involvement by lymphoma. Imaging of additional areas could of course reveal unexpected focal deposits of lymphoma.

Magnetic resonance imaging is unlikely to replace bone marrow biopsy in the management of patients with lymphoma, particularly as important phenotypic and cytogenetic information may be derived from biopsies. MRI may, however, have a complementary role. Abnormal results on MRI may alert the physician to probable bone marrow involvement and suggest optimal sites for confirmatory biopsy. Further studies are required to evaluate the accuracy of MRI in monitoring response to therapy. Serial bone marrow $T_1$ measurement could potentially reduce the number of repeat bone marrow biopsies required during follow up.

### References

BONADONNA, G., CASTELLANI, R., NARDUZZI, C., SPINELLI, P. & RILKE, F. (1979) Pathological staging in adult previously untreated non-Hodgkin's lymphomas. In *Lymphoid Neoplasms II. Clinical and Therapeutic Aspects*, Mathe *et al.* (ed) Springer-Verlag, New York.

BRUNNING, R.D., BLOOMFIELD, C.D., McKENNA, R.W. & PETERSON, L. (1975). Bilateral trephine bone marrow biopsies in lymphoma and other neoplastic diseases. *Ann. Int. Med.*, **83**, 365.

CHABNER, B.A., JOHNSON, R.E., DeVITA, V.T. & 4 others (1977). Sequential staging in non-Hodgkin's lymphoma. *Cancer Treat. Rep.*, **61**, 993.

COHEN, M.D., KLATTRE, E.C., BAEHNER, R. & 11 others (1984). Magnetic resonance imaging of bone marrow disease in children. *Radiology*, **151**, 715.

GRANN, V., POOL, J.L. & MAYER, K. (1966). Comparative study of bone marrow aspiration and biopsy in patients with neoplastic disease. *Cancer*, **19**, 1898.

JONES, S.E., ROSENBERG, S.A. & KAPLAN, H.S. (1972). Non Hodgkin's lymphomas I. Bone marrow involvement. *Cancer*, **29**, 954.

KRICUN, M.R. (1985). Red-yellow marrow conversion: Its effect on the location of some solitary bone lesions. *Skeletal Radiol.*, **14**, 10.

McKINSTRY, C.S., JONES, L., STEINER, R.E. & BYDDER, G.M. (1986). NMR imaging of the bone marrow in leukaemia treated by bone marrow transplantation. *Proc. Soc. Magnetic Resonance Med.*, **2**, 577.

MOORE, S., GOODING, C., EHMEN, R. & BRASCH, R. (1985). Intensity measurement of the marrow in patients with acute lymphocytic leukaemia. *Proc. Soc. Magnetic Resonance Med.*, **2**, 1193.

OLSON, D.O., SHIELDS, A.F., SCHEURICH, C.J., PORTER, B.A. & MOSS, A.A. (1986). Magnetic resonance imaging of the bone marrow in patients with leukaemia, aplastic anaemia and lymphoma. *Invest. Radiol.*, **21**, 540.

REDPATH, T.W. (1982). Calibration of the Aberdeen NMR imager for proton spin-lattice relation time measurements *in vivo*. *Phys. Med. Biol.*, **27**, 1057.

RICHARDS, M.A., GREGORY, W.M., WEBB, J.A.W., JEWELL, S.E. & REZNEK, R.H. (1987). Reproducibility of spin lattice relaxation time ($T_1$) measurement using an 0.08 Tesla magnetic resonance imager. *Br. J. Radiol.*, **60**, 241.

RICHARDS, M.A., WEBB, J.A.W., JEWELL, S.E., GREGORY, W.M. & REZNEK, R.H. (1988). *In vivo* measurement of spin lattice relaxation time ($T_1$) of bone marrow in healthy volunteers: The effects of age and sex. *Br. J. Radiol.*, **61**, 30.

ROSENBERG, S.A. (1971). Hodgkin's disease of the bone marrow. *Cancer Res.*, **31**, 1733.

SHIELDS, A.F., PORTER, B.A., CHURCHLEY, S., OLSON, D.O., APPELBAUM, F.R. & THOMAS, E.D. (1987). The detection of bone marrow involvement by lymphoma using magnetic resonance imaging. *J. Clin. Oncol.*, **5**, 225.

SHILLINGFORD, J.P. (1950). The red bone marrow in heart failure. *J. Clin. Path.*, **3**, 24.

THOMSEN, C., GRUNDTVIG, P., KARLE, H., HENRIKSEN, O. & CHRISTOFFERSEN, P. (1986). *In vivo* estimation of relaxation processes by magnetic resonance in the bone marrow in patients with acute leukaemia. *Proc. Soc. Magnetic Resonance Med.*, **2**, 277.

VINCIGUERRA, V. & SILVER, R.T. (1973). The importance of bone marrow biopsy in the staging of patients with lymphosarcoma. *Blood*, **41**, 913.